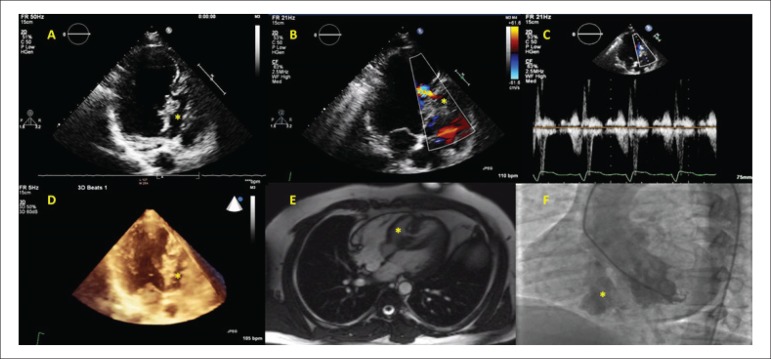# Congenital Muscular Interventricular Septal Malformation with Complex Anatomical Features

**DOI:** 10.5935/abc.20160045

**Published:** 2016-04

**Authors:** Zafer ışılak, Mehmet Uzun, Ejder Kardeşoğlu, Ömer Uz, Uğur Küçük

**Affiliations:** Department of Cardiology, Gulhane Military Medical Academy, Haydarpasa Training Hospital, Istanbul - Turquia

**Keywords:** Heart Defects, Congenital, Ventricular Septum, Heart Septal Defects, Ventricular

A 21 year-old male patient was admitted with symptoms of exertional dyspnea and palpitation. The physical examination revealed a grade 3/6 systolic murmur, best heard over the left 3-4th intercostal space. Transthoracic echocardiography disclosed a separate chamber (asterisk) in the interventricular septum. The apical portion of the chamber consisted of muscle tissue and the basal portion consisted of membranous and aneurysmatic tissue (Panel A). There was a muscular "tunnel-like" structure connecting the left ventricle and chamber at mid ventricular level. The color and continuous wave Doppler imaging revealed a bidirectional flow across the passage (Panel B, C).

The patient underwent three dimensional transthoracic echocardiographic examinations, which revealed two separate septa. Between these septa there was a third chamber (asterisk). It was connected to both left (via the tunnel at the muscular septum) and right ventricles (via the defect in the membranous septal aneurysm). The membranous septal aneurysm was separated from the left ventricle by a thin membrane, without any passage across it (Panel D). MRI findings were consistent with echocardiographic images (Panel E). Ventriculography and coronary angiography were performed. The coronary arteries were normal; the left ventriculography showed the muscular and membranous/aneurysmatic portions of the malformation (Panel F). The patient was advised to undergo a surgical procedure, but he refused and was discharged with recommendations about control visits.

## Figures and Tables

**Figure:**